# Supplementary material for: Mapping the evolution of fertility support policies in China: A content and instrumental analysis
Source: PLoS One. 2025 Oct 9;20(10):e0332137. doi: 10.1371/journal.pone.0332137 (PMC12510515; doi:10.1371/journal.pone.0332137)
Supplement: S1 Appendix — (ZIP) [file pone.0332137.s001.zip › S1 Appendix. 226 original policy documents/212-第十届全国人民代表大会第三次会议关于2004年国民经济和社会发展计划执行情况与2005年国民经济和社会发展计划的决议(FBM-CLI-1-175094).doc]

第十届全国人民代表大会第三次会议关于2004年国民经济和社会发展计划执行情况与2005年国民经济和社会发展计划的决议

**发布部门：** [全国人民代表大会](https://www.pkulaw.com/law/chl?Aggs.IssueDepartment=101)

**发布日期：**2005.03.14

**实施日期：**2005.03.14

**时效性：** [现行有效](https://www.pkulaw.com/law/chl?Aggs.TimelinessDic=01)

**效力级别：** [工作文件](https://www.pkulaw.com/law/chl?Aggs.EffectivenessDic=XA0105)

**法规类别：** [人大议事](https://www.pkulaw.com/law/chl?Aggs.Category=00106)

第十届全国人民代表大会第三次会议关于2004年国民经济和社会发展计划执行情况与2005年国民经济和社会发展计划的决议
（2005年3月14日第十届全国人民代表大会第三次会议通过）

　　第十届全国人民代表大会第三次会议审查了国务院提出的《关于2004年国民经济和社会发展计划执行情况与2005年国民经济和社会发展计划草案的报告》及2005年国民经济和社会发展计划草案。会议同意全国人民代表大会财政经济委员会的审查结果报告，决定批准《关于2004年国民经济和社会发展计划执行情况与2005年国民经济和社会发展计划草案的报告》，批准2005年国民经济和社会发展计划。

关于2004年国民经济和社会发展计划
执行情况与2005年国民经济和
社会发展计划草案的报告
--2005年3月5日在第十届全国人民代表大会第三次会议上
国家发展和改革委员会

各位代表：
　　受国务院委托，现将2004年国民经济和社会发展计划执行情况与2005年国民经济和社会发展计划草案提请十届全国人大三次会议审议，并请全国政协各位委员提出意见。
　　**一、**2004年国民经济和社会发展计划执行情况
　　2004年，全国各族人民在中国共产党的领导下，认真实施十届全国人大二次会议批准的国民经济和社会发展计划，树立和落实科学发展观，加强和改善宏观调控，同心同德，奋发图强，促进国民经济持续快速协调健康发展和社会全面进步。一方面，经济生活中的突出矛盾得到缓解，不健康不稳定因素得到抑制；另一方面，国民经济保持了增长比较快、效益比较好、活力比较强的良好势头。计划执行情况总的是好的。全年国内生产总值达到136515亿元，增长9.5%，高于年初的预期目标，主要是由于投资增幅较高和出口增长强劲的带动。城镇新增就业980万人，比预期目标多增加80万人；城镇登记失业率4.2%，比预期目标降低0.5个百分点。受粮价上涨和国际市场石油价格大幅度上涨等因素的影响，居民消费价格总水平上涨3.9%，略高于3%左右的预期目标。随着世界经济持续复苏和国内经济增长加快，外贸进出口总额增长35.7%。
　　(一)经济平稳较快增长，效益明显提高。针对一季度固定资产投资增长过快等突出问题，中央果断决策，及时采取一系列加强和改善宏观调控的措施，遏制了投资过快增长的势头，固定资产投资增幅逐季回落，消费需求有所回升。全社会固定资产投资总额70073亿元，增长25.8%，增幅比上年回落1.9个百分点，其中四季度增幅为22.6%，比一季度回落20.4个百分点。全社会消费品零售总额53950亿元，增长13.3%，扣除价格因素，实际增长10.2%，比上年提高1个百分点。全国财政收入达到26356亿元，增长21.4%。规模以上工业企业实现利润11342亿元，增长38.1%。
　　(二)产业结构调整积极推进，薄弱环节得到加强。国内生产总值中，第一产业完成增加值20744亿元，增长6.3%；第二产业72387亿元，增长11.1%；第三产业43384亿元，增长8.3%。
　　粮食生产出现重要转机，农业结构调整继续推进。粮食播种面积扭转了连续5年下滑的趋势，恢复到10161万公顷，比上年增长2%左右。粮食总产量达到4695亿公斤，增产388亿公斤。优势农产品产业带建设得到加强，优质粮食和蔬菜水果品种比重上升，棉花获得较大丰收，畜牧业、水产业发展加快。
　　能源建设得到加强，交通基础设施进一步完善。陕京二线输气管道、广东液化天然气项目一期、国家石油储备基地、煤矿安全改造、大型煤炭基地、农网改造等一批重大能源项目建设进展顺利。新增煤炭生产能力1.2亿吨。新增发电装机容量5055万千瓦。铁路新线投产1433公里。新增公路通车里程4.6万公里，其中高速公路4476公里。新增港口吞吐能力1.2亿吨。广州、重庆、青岛机场新改建工程相继竣工，一批支线机场建成投入使用。
　　传统产业升级加快，高技术产业快速增长。纺织行业差别化纤维用量比重达到28%，比上年提高3个百分点。新型干法水泥产量比重达到32.5%，提高7.5个百分点。钢材板带比达到33.7%，提高1.6个百分点。规模以上高技术企业完成增加值增长23.1%，高出规模以上工业增幅6.4个百分点，生物、集成电路、软件等一批对经济发展有重大带动作用的高技术产业正在形成。
　　(三)西部大开发取得新进展，东北地区等老工业基地振兴开局良好。西部地区基础设施建设成效明显。2004年，安排新开工重点工程10项，总投资约800亿元。西气东输管道工程如期实现全线商业运营。西电东送全年新开工装机容量800万千瓦，形成2000万千瓦以上的总供给能力。青藏铁路新铺轨396公里。兴义、拉萨、和田、延安等新建和扩建机场投入使用。生态环境继续改善。完成退耕还林66.7万公顷、荒山荒地造林325.5万公顷，治理严重退化草原560万公顷。东北地区等老工业基地调整改造步伐加快，提升企业技术创新能力的高技术项目实施顺利，采煤沉陷区的治理全面展开。东、中部地区在宏观调控中继续推进特色经济发展，结构调整步伐加快。
　　(四)经济体制改革进一步深化，对外开放不断扩大。粮食收购市场全面放开。大型国有企业股份制改革步伐加快，国有资产监督管理体制框架初步建立。中国银行和中国建设银行股份制改革试点顺利推进，农村信用社改革试点范围进一步扩大，利率市场化改革取得重要进展。投资体制改革方案开始实施。取消了除烟叶以外的农业特产税，减免了农业税。启动了东北老工业基地增值税转型改革试点。出口退税机制改革全面实施。行政审批制度改革继续深化，再次取消和调整行政审批项目495项。新型农村合作医疗试点和城市医疗服务体制改革工作继续推进。文化体制改革进展顺利。
　　对外贸易跃上新台阶。全年外贸进出口总额11548亿美元。其中，出口5934亿美元，增长35.4%；进口5614亿美元，增长36%；进出口贸易顺差320亿美元。出口商品结构继续改善，机电产品、高新技术产品出口快速增长。年末外汇储备达到6099亿美元。利用外资规模扩大、质量提高。全年实际利用外商直接投资606亿美元，增长13.3%。服务领域投资继续增长，跨国公司在华设立研发中心和地区总部数量明显增加。实施“走出去”战略取得新进展。内地与香港、澳门建立更紧密经贸关系安排顺利推进。
　　(五)科技、教育、文化、卫生等各项事业加快发展，生态建设和环境保护迈出新步伐。基础研究和战略高技术研究又取得一批重大成果。国家创新体系、科技基础设施和产业创新能力建设得到加强。组织实施下一代互联网、第三代移动通信、生物技术、现代农业、新材料等高技术产业化专项，生物、软件等领域建成了一批国家高技术产业基地，重大技术装备和重大产业技术研发工作稳步推进，自主研制和国际合作生产支线飞机取得重要进展。教育事业加快发展。“两基”人口覆盖率不断提高，高中阶段教育规模扩大，中等职业技术教育招生规模继续回升，各类高等教育在学人数超过2000万人。
　　公共卫生体系建设和人口、计划生育工作得到加强。9个省级、241个地市级和1410个县级疾病预防控制中心基本建成，290所紧急救援中心和2074所传染病治疗机构建设项目陆续开工，部分项目建成使用。中西部地区艾滋病、血吸虫病重点防治县和合作医疗试点县的3787个乡镇卫生院设施条件得到改善。人口自然增长率5.87‰。
　　城乡文化设施建设取得新进展。县级图书馆、文化馆和农村广播电影电视设施建设继续加强。国家重大文化项目建设和自然文化遗产保护力度加大。全民健身活动广泛开展，竞技体育成绩显著。奥运会场馆设施建设稳步推进。
　　环境保护和生态建设力度加大。“三河三湖”、渤海等重点流域、海域水污染防治工程加紧实施，部分水域水质有所改善。三峡库区地质灾害，库区及影响区的城镇生活污水、工业废水、危险废物以及火电厂二氧化硫污染治理进一步加强。南水北调东线治污步伐加快。城市污水处理率和工业固体废物综合利用率又有提高。天然林资源保护、“三北”和长江中下游重点防护林、野生动植物保护和自然保护区等工程建设成效明显。
　　(六)就业和社会保障工作取得新成绩，人民生活继续改善。城镇下岗失业人员再就业510万人。企业退休人员基本养老金标准和部分地区城镇居民最低生活保障标准得到提高。城镇基本养老、基本医疗、失业、工伤保险参保人数分别达到1.63亿人、1.24亿人、1.06亿人和6823万人。城镇居民人均可支配收入9422元，实际增长7.7%；农民人均纯收入2936元，实际增长6.8%。年末农村绝对贫困人口和低收入人口分别比上年末减少290万人和640万人。
　　在充分肯定成绩的同时，要清醒地看到，加强和改善宏观调控的成效还是阶段性的，经济运行中的突出矛盾和问题虽有所缓解，但还没有根本解决，保持经济平稳较快发展良好态势面临诸多挑战。一是农业基础设施仍很薄弱，继续扩大粮食播种面积和提高单位面积产量受到很大制约，气候条件存在不确定性，保持粮食增产和农民增收好势头的难度加大。二是固定资产投资在建规模依然偏大，新开工项目较多，投资扩张的冲动较强，投资需求膨胀还有可能反弹。三是资源环境约束矛盾突出，煤电油运仍相当紧张，价格上涨的压力较大。一些地方环境污染还相当严重。四是农村教育、卫生、文化等社会事业远不能适应农村发展和人民生活水平提高的需要。五是就业形势不容乐观，部分社会成员之间收入差距过大，一些低收入群众生活比较困难。六是经济社会发展中的深层次矛盾尚未根本消除。经济体制不完善，经济结构不合理，经济增长方式粗放。对这些问题，要积极采取措施，努力加以解决。

　　**二、**2005年经济社会发展的总体要求和预期目标
　　2005年是巩固和发展宏观调控成果、保持经济社会发展良好态势的关键一年，也是全面完成“十五”计划、为“十一五”发展打好基础的重要一年。今年经济工作的总体要求是：以邓小平理论和“三个代表”重要思想为指导，认真贯彻中国共产党第十六次全国代表大会和十六届三中、四中全会精神，全面落实科学发展观，坚持发展这个党执政兴国的第一要务，进一步提高驾驭社会主义市场经济的能力，加强和改善宏观调控，着力推进改革开放，加快调整经济结构、转变经济增长方式，更好地利用重要战略机遇期，实现经济社会全面协调可持续发展。
　　2005年国民经济和社会发展的主要预期目标为：
　　--国内生产总值增长8%左右。主要考虑是：第一，从需要看，在优化结构、提高效益和资源环境可以承受的前提下，保持较快的经济增长速度，对于用好重要战略机遇期、增强综合国力、实现全面建设小康社会目标是完全必要的，也有利于进一步扩大就业、推进各项改革、维护社会稳定。第二，从可能看，近两年我国能源、原材料和交通运输供给能力大幅度增加，但煤电油运供求紧张的矛盾却依然突出。在我国经济规模已经较大而经济增长方式粗放的情况下，如果增长速度过高，对资源供给和环境保护的压力会更大，经济运行会绷得更紧，矛盾会更加突出。第三，从国家宏观调控的意图看，适当的增长速度有利于引导各方面按照科学发展观的要求，克服盲目攀比增长速度倾向，真正把工作重点放到调整结构、深化改革和转变增长方式上来。8%左右是全国经济增长预期目标，各地区要从实际出发，合理确定本地区的预期目标。
　　--城镇新增就业900万人，城镇登记失业率控制在4.6%。今年城镇新增劳动力、复员转业军人、高校毕业生等需要就业人员1100万，现有失业和下岗人员1300万，还有大批农村劳动力需要向城镇转移，就业压力仍然很大。根据近年来经济增长与提供就业岗位的关系测算，国家通过实施积极的就业政策，预计今年可以新增900万个就业岗位。考虑到今年要基本完成国有企业下岗职工基本生活保障向失业保险并轨，预计年末城镇登记失业率会比上年有所提高。
　　--居民消费价格总水平涨幅控制在4%。今年仍存在一些推动居民消费价格上涨的因素：一是煤电油运供求关系依然偏紧，生产资料价格持续高位运行，推动下游产品价格上涨的压力较大，并可能逐步传导到居民消费品价格上来。二是国际市场石油和原材料价格较高，会拉动国内市场价格上涨。三是去年一些地方政府调价项目推迟出台，公用事业和服务领域积累了一些价格矛盾。四是资金、土地等生产要素改革的推进和劳动力工资水平的提高，也会在一定程度上推动居民消费价格水平的上升。与此同时，国家将继续加强和改善宏观调控，合理调控货币信贷总量，控制固定资产投资过快增长，有利于抑制生产资料价格上涨；上年价格上涨对今年的滞后影响也相对较少。综合分析影响价格上升和下降的因素，今年居民消费价格总水平涨幅预期与上年实际涨幅大体相当。
　　--外贸进出口总额增长15%，进出口基本平衡。2005年世界经济和贸易仍将保持增长态势，外贸经营权全面放开，将给我国外贸出口带来新的活力，有利于扩大出口；国内经济继续较快增长，进口需求也将不断扩大，今年外贸进出口仍会保持较快增长。但是，保持上年那样的高速增长也有不少困难，主要是国际油价居高不下，生产资料价格上涨，带动企业生产成本增加；近年来出口持续大幅增长，基数较高，国际贸易摩擦增加。同时，这个预期指标也有利于引导企业加快转变外贸增长方式，优化进出口商品结构，提高外贸增长的质量和水平。
　　--城乡居民收入进一步增加。今年城镇居民人均可支配收入和农民人均纯收入预期实际分别增长6%和5%左右。主要考虑是：今年国民经济保持较快增长，国家继续采取措施促进农业增产和农民增收，企业经济效益可望继续提高，国家加大社会保障和扶贫工作力度，这些都有利于增加城乡居民收入。另一方面，增加居民收入特别是农民收入也面临很多困难和不确定因素。居民收入水平持续提高为扩大消费奠定了基础，加上消费结构升级和消费环境不断改善，今年消费需求将继续平稳较快增长，全社会消费品零售总额预期增长12.5%。
　　--科技、教育、文化、卫生、体育事业加快发展。认真落实科教兴国和人才强国战略，采取有效措施，围绕提高国民经济整体素质、自主创新能力和竞争力，进一步增加对科技教育的投入。重大科技基础设施建设和重大产业技术开发取得新的进展，研究与试验发展经费支出占国内生产总值的比例力争达到1.5%。素质教育全面推进，教育结构不断优化，农村教育特别是西部地区“两基”攻坚计划取得明显进展，高中阶段教育毛入学率达到50%，普通高校计划招生475万人，研究生37万人。疾病预防控制体系全面建成，医疗救治体系建设基本完成，新型农村合作医疗试点稳步推进。农村自来水普及率继续提高。县级图书馆、文化馆建设任务全面完成，广播影视覆盖面进一步扩大，满足人民群众精神文化生活需求的其他各项社会事业继续发展。
　　--资源利用效率有所提高，生态建设和环境保护取得新进展。以节能、节水、节地、节材为重点的节能降耗工作取得新成效，冶金、有色、煤炭、化工、建材、轻工等重点行业和矿产资源节约利用、再生资源回收利用、绿色再制造等重点领域在发展循环经济上迈出新步伐。力争使每万元国内生产总值能耗、部分主要污染物排放量有所下降，工业固体废物综合利用率、城市污水处理率进一步提高，水土流失和“三化”草地治理面积、矿山生态恢复面积继续增加。
　　--人口继续保持低生育水平，出生人口素质进一步提高。人口自然增长率控制在7‰以内。这个指标高于上年的实际水平，主要原因是：我国人口总量很大，稳定低生育水平的任务仍很艰巨；人口年龄结构因素导致的生育旺盛期妇女人数增多，对生育率回升构成现实压力；流动人口计划生育工作难度依然较大；农村地区和中西部贫困地区稳定低生育水平还相当困难。同时，针对部分地区出生人口缺陷发生率较高的情况，进一步做好优生优育工作，提高出生缺陷干预能力和生殖健康服务水平。
　　在社会主义市场经济条件下，经济和社会发展预期目标是导向性的，是国家向社会传递的宏观调控意图。预期目标的确定，要兼顾需要和可能，适当留有余地。同时，预期目标也是动态的，可以随着经济运行环境和发展趋势的变化进行调整。
　　实现今年各项经济和社会发展的预期目标，保持国民经济平稳较快发展，最根本的是要以科学发展观统领经济社会发展全局，并切实贯穿于经济社会发展和改革开放的各个领域，努力做到五个坚持：一是坚持加强和改善宏观调控，更加注重区别对待、有保有压，进一步消除经济运行中的不健康不稳定因素，把各方面加快发展的积极性保护好、引导好、发挥好；二是坚持以改革开放为动力，注重用改革的办法解决阻碍经济社会发展的体制性、机制性问题，力争在一些重点领域和关键环节实现新的突破；三是坚持以结构调整为主线，围绕提高国民经济整体素质和竞争力，推动技术进步，促进产业结构优化升级；四是坚持走新型工业化道路，加快推进经济增长方式转变，积极发展循环经济，努力建设节约型社会；五是坚持以人为本，认真解决关系群众切身利益的问题，不断改善城乡居民生活质量，努力构建社会主义和谐社会。

　　**三、**2005年经济社会发展的主要任务和措施
　　实现今年国民经济和社会发展的预期目标，要着力抓好以下工作。
　　(一)坚持加强和改善宏观调控，保持经济平稳较快发展。把握好调控的重点和力度，充分发挥市场机制的作用，更加注重运用经济手段和法律手段，巩固和发展宏观调控成果。
　　正确把握宏观经济政策取向，实行稳健的财政政策和货币政策。(1)适当减少财政赤字，着力调整财政支出结构。2005年，全国财政预算收入29255亿元，支出32255亿元，中央财政赤字控制在3000亿元，比2004年预算减少198亿元，赤字占国内生产总值的比重进一步降低。依法加强税收征管，确保财政收入稳定增长。严格控制一般性支出增长，加大对中西部地区特别是农村教育、卫生、文化和县乡基层政权正常运转等的转移支付力度，加大对解决“三农”问题、促进社会发展和区域协调、扩大就业、完善社会保障制度、保障低收入群体生活等方面的投入力度，加大对重要矿产资源开发的支持力度。(2)适当减少长期建设国债发行规模，继续调整国债资金投向结构。2005年拟发行长期建设国债800亿元，比2004年减少300亿元；增加中央预算内经常性建设资金100亿元。国债资金主要用于：加强农业基础设施和重大水利项目建设，支持粮食生产；加大对基础教育、公共卫生体系、生态建设和环境保护、资源节约和发展循环经济等方面的投入；支持西部大开发、东北地区等老工业基地调整改造和中部崛起的重点项目；保证在建国债项目按期完成，主要包括青藏铁路、南水北调、治淮工程、病险水库除险加固、农村饮水安全、县际和农村公路、采煤沉陷区治理等在建项目。继续加强建设项目的稽察监管，进一步提高投资效益。(3)灵活运用货币政策工具，合理调控货币信贷总量。2005年广义货币供应量M2和狭义货币供应量M1预期增长15%。加强和改进对商业银行的窗口指导，引导金融机构优化信贷结构，改进金融服务。控制中长期贷款增长幅度，严格控制对不符合国家产业政策特别是高耗能、高污染项目的贷款。支持符合国家产业政策和市场准入条件的项目建设，支持有市场、有效益、能增加就业的企业流动资金贷款需求。加强金融监管，积极稳妥地处置各类金融隐患，防范和化解金融风险。
　　加强各项宏观经济政策的协调配合，继续控制固定资产投资过快增长。2005年全社会固定资产投资总规模预期增长16%。进一步加强产业政策、信贷政策、土地政策等的协调配合，继续把好土地审批和信贷投放两个闸门。加快制定、完善并认真实施重点行业的产业政策、专项规划和能耗、物耗、环保、安全等市场准入标准。落实已确定的各项调控政策，防止钢铁、水泥、电解铝、电石、焦炭、铁合金等行业投资反弹。抓紧解决火电建设规模过大和违规建设问题。继续做好投资项目清理的后续工作。着力优化投资结构，引导社会资金投向需要加快发展的薄弱环节。
　　继续加强经济运行调节，缓解煤电油运供求紧张的矛盾。(1)在确保安全生产的前提下，组织和引导企业努力增加有效供给。重点支持大型煤炭基地建设，加大运煤铁路和北方下水港口等煤炭运输通道改扩建力度，继续支持煤矿安全生产更新改造。加快煤炭液化、石油天然气开发、天然气发电、可再生能源等重点工程建设，合理发展水电。加强跨区域电网建设。实施煤电价格联动机制，鼓励煤矿与用户签订中长期合同。(2)继续加强电力需求侧管理。贯彻有保有限原则，落实有序供电、合理用电方案。完善峰谷分时电价和差别电价政策，促进节约用电。(3)合理安排运力，优先满足粮食、煤炭、石油、化肥等重点物资的运输需求。
　　进一步做好价格工作，保持价格总水平基本稳定。继续实行居民消费价格指数月度通报制度。根据各地区经济发展状况和群众承受能力，把握好政府调整公共产品和服务价格的时机和力度。完善价格监测体系以及价格异常波动的预警和应急机制。加强煤电油运市场价格巡查和涉农、医药价格以及教育收费的监督检查，加大对价格欺诈、哄抬价格等不法行为的整治力度，认真清理和整顿房地产业乱收费。
　　(二)加强农业特别是粮食生产，努力增加农民收入。坚持统筹城乡发展，以工促农，以城带乡，继续贯彻“多予、少取、放活”方针，采取综合措施，努力实现粮食稳定增产，农业持续增效，农民继续增收。(1)完善和强化对农业的扶持政策。进一步大范围、大幅度减免农业税。592个国家扶贫开发工作重点县免征农业税。全部免征牧业税。中央财政安排专项资金，加大对粮食主产县特别是财政困难县乡的转移支付力度。完善对种粮农民的直接补贴制度，增加良种补贴和农机具购置补贴。建立粮食主产区与主销区之间的利益协调机制。(2)切实加强农业综合生产能力建设。实行最严格的耕地保护制度，确保基本农田总量不减少、用途不改变、质量不下降，努力扩大粮食播种面积。认真落实农村土地承包政策，完善土地征收征用制度。加大投入力度，推进优质粮食产业工程和大型商品粮生产基地建设，加快中低产田改造，加大对小型农田水利建设的支持力度，扩大农村“六小工程”建设范围。加快灌区节水改造，发展旱作节水农业。继续支持种子工程、植保工程和沃土工程建设，加快推进农村标准化和农产品质量安全检验检测体系建设。(3)加快农业和农村经济结构调整。通过政策指导、规划引导和项目带动，推进农业区域化布局、专业化生产、产业化经营，大力发展特色农业。大幅度增加对农业科研的投入，加强农业科技创新能力和技术推广体系建设。加大粮食优良品种繁育和推广力度，继续实施畜禽水产良种工程，建立健全动物疫病防治体系，大力发展畜牧业。积极发展农村二、三产业，支持农产品加工业，完善农产品流通体系。(4)稳定涉农价格。在主产区继续对短缺的重点粮食品种实行最低收购价政策。加快建设国家粮食安全预警系统，切实做好粮食进出口调控和储备调节，努力使粮食价格保持在合理水平。落实促进化肥等农业生产资料生产和稳定供应的各项政策，防止价格过快上涨。(5)拓展农民增收渠道。推进城镇化进程，促进县域经济发展，扩大农民就业门路。加强对农村劳动力的职业技能培训。进一步改善农民进城务工环境和劳动条件，建立健全防止拖欠农民工工资的机制。继续做好以工代赈工作，因地制宜地实施生态移民，提高扶贫开发成效。
　　(三)着力推进结构调整，加快产业升级。以加强和改善宏观调控为契机，按照走新型工业化道路的要求，加大产业结构调整力度。(1)加快振兴装备制造业。抓紧出台鼓励政策。依托国家重点工程，开发具有自主知识产权的核心技术。重点抓好核电机组、超超临界发电机组、直流输电设备、清洁高效电力设备、新型轨道交通车辆以及大型石化和大型薄板冷热连轧成套设备等重大技术装备的本地化制造，提高以数控机床和自动化控制系统为代表的基础技术装备、新型农业技术装备及其他节能、节水、节材、环保型设备的设计、研发和成套能力，培育和发展知名品牌。加快环渤海湾、长江口、珠江口等重要造船基础设施和重点船用配套项目建设。努力增强自主创新能力和核心竞争力，支持企业开发具有自主知识产权的关键技术，重点提高重大装备和基础软件的自主开发能力。(2)大力发展高新技术产业。积极推进若干重大领域关键技术创新。做大做强信息产业，培育生物产业，加快发展航空航天产业，继续组织实施海水淡化、汽车电子等重点产业化专项，积极引导和扶持新材料、新能源和先进环保产业。新建清洁生产技术、煤炭安全采掘与高效利用等国家工程研究中心。加快组织研制高速铁路、大型高技术船舶等重大技术装备。积极稳妥地推进金税、金关、金财、金农、金质等电子政务系统建设。大力发展电子商务，加快电子信息技术在外贸、石化、冶金、机械等重点领域的应用，进一步推进大型骨干企业的信息化建设。建立网络与信息安全保障体系。(3)促进传统产业优化升级。抓紧发布和实施钢铁、电解铝、水泥、化肥等行业产业政策和规划。钢铁行业，支持大型钢铁企业联合重组和建立精品钢材生产基地。电解铝行业，在调控总量的前提下，结合淘汰落后技术设备，适度建设有稳定氧化铝原料供应和电力保障的项目，加快现有大型铝基地建设。水泥行业，继续发展符合规模要求的新型干法水泥，鼓励大型企业跨地区发展、重组和联合。加强可再生能源开发利用，做好车用乙醇汽油的试点工作。清理整顿破坏、浪费资源和污染环境的项目。(4)加快发展服务业。优先发展现代金融、现代物流和信息服务等新兴服务业。积极发展旅游、文化、体育等需求潜力大的行业，形成新的增长点。加快发展咨询、法律服务、科技服务等中介服务业。规范和提升传统服务业，鼓励和扶持社区服务业发展。
　　(四)积极转变经济增长方式，大力推进资源节约和环境保护。(1)突出抓好节能、节水、节地、节材。落实好《节能中长期专项规划》，抓紧组织制定《节约石油管理办法》和《资源综合利用条例》，修订《[节约用电管理办法](https://www.pkulaw.com/chl/a08ee3f10270655bbdfb.html)》。抓紧制定和完善用能产品能效和重点行业取水定额标准，实行高能耗产品淘汰制度，制订并发布节能、节水技术政策大纲，支持开发和应用节能降耗和综合利用新技术、新工艺、新设备。以降低能源消耗特别是石油消耗为重点，加快利用先进适用技术改造传统产业，重点抓好冶金、有色、石化、建材、造纸等行业节能、节水技术改造，研究提出发展节能环保型汽车、新型墙体材料和节能住宅的相关政策。(2)加强矿产资源开发规划和管理。改革资源开采体制，抓紧完善资源开发利用补偿机制和生态环境恢复补偿机制，加强能源矿产的风险勘查与潜力评价。坚决制止能源和其他矿产资源开发中的滥采乱挖、严重浪费和破坏生态现象。加快开发应用新能源和可再生能源，支持利用煤矸石、垃圾焚烧和秸秆发电。加强油页岩的调查评价与开发利用。(3)加快发展循环经济。制定发展循环经济的战略目标、评价指标体系和政策措施。支持开发和推广资源节约和替代技术、回收处理技术、绿色再制造技术、零排放技术。组织实施循环经济示范试点，支持一批节能、节水、资源综合利用、发展循环经济的重大项目。广泛开展促进循环经济发展和建设节约型社会的宣传教育培训活动。(4)切实加强环境保护工作。继续推行清洁生产，制定重点行业清洁生产评价指标体系。控制污染物排放总量。加强火电厂二氧化硫治理工作，大力推进清洁电源建设，发展洁净煤技术。加大“三河三湖”、渤海、三峡库区及其上游、南水北调沿线等重点区域、重大工程污染防治力度，将黄河、松花江纳入重点流域治理范围。编制和实施淮河流域工业废水治理规划。推动113个环境保护重点城市环境质量的改善。建立和完善城市垃圾和污水处理收费制度，加快推进城市生活垃圾、生活污水处理和再生利用设施建设。
　　(五)充分发挥各地区的优势和积极性，促进区域协调发展。积极推动东中西部地区优势互补、良性互动、共同发展。(1)进一步推进西部大开发。继续抓好交通、能源、水利等重大项目和农村基础设施建设。巩固退耕还林成果，加快退牧还草进度。加强能源、矿产加工业、石化和煤化工基地建设，促进旅游业和特色农牧产品加工业发展。支持西部地区社会事业加快发展。增加中央财政对西部地区的一般性转移支付和专项补助资金，抓紧建立西部开发长期稳定的专项资金渠道。加强人才队伍和法制建设。抓紧起草《西部开发促进法》，研究提出西部地区人才队伍建设的政策措施。(2)继续实施东北地区等老工业基地振兴战略。加快体制机制创新。大力发展现代农业，巩固东北全国商品粮基地的战略地位，推进东北钢铁、石化、装备等产业升级和重点地区、重点优势产业以及重点企业的调整改造及资产重组，研究建立资源开发补偿机制和衰退产业援助机制，促进资源型城市经济转型。(3)抓紧制定促进中部崛起的政策措施，支持农业特别是粮食主产区建设，加强交通、能源、重要原材料基地建设，加快发展有竞争力的制造业和高新技术产业，开拓中部地区大市场，发展大流通。(4)引导东部地区加快发展，进一步提高经济整体素质和国际竞争力，提升本地区城乡、区域、经济社会、人与自然协调发展水平。(5)支持和促进老少边穷地区加快发展，在财政转移支付、资金投入和重大建设项目安排等方面给予更多的扶持。
　　(六)积极推进各项改革，为经济社会发展增添新的动力。围绕解决影响全局的深层次矛盾和问题，加快推进经济市场化改革进程，力争在一些重点领域和关键环节取得新的突破。(1)进一步推进国有大型企业股份制改革，健全公司法人治理结构，转换企业经营机制。积极培育和发展具有国际竞争力的大企业集团。推进企业分离办社会，继续做好国有企业政策性关闭破产工作。规范国有企业改制和产权转让，促进国有资产有序流动，防止国有资产流失，维护职工合法权益。(2)抓紧健全国有资产监督管理体制，建立和完善激励、约束机制，在中央企业全面实行年度经营业绩责任制和任期经营业绩考核责任制，加快建立国有资本经营预算制度。(3)完善电信、电力、民航等行业改革措施，适时出台邮政体制改革方案，研究提出铁路体制改革方案，推进供水、供气、污水和垃圾处理等市政公用事业的市场化进程。(4)认真贯彻落实《[国务院关于鼓励支持和引导个体私营等非公有制经济发展的若干意见](https://www.pkulaw.com/chl/c89aff4f1da83473bdfb.html)》(国发[2005]3号)，抓紧研究制定相关配套措施，鼓励非公有制经济参与国有企业改组改造，进入基础设施、公用事业及其他行业和领域。继续贯彻实施《[中小企业促进法](https://www.pkulaw.com/chl/b10fbeb3424189d7bdfb.html)》。(5)积极推进财税体制改革。完善省以下财政体制，加大转移支付力度。在总结东北地区试点经验的基础上，研究在全国推开增值税转型的方案。(6)继续抓好国有商业银行股份制改革，加快建立健全商业银行风险管理的长效机制。推进股份制商业银行、政策性银行改革。着力解决农村信用社改革中的产权制度和管理体制问题，规范中小金融机构发展。稳步推进利率市场化改革，完善人民币汇率形成机制，保持人民币汇率在合理、均衡水平上基本稳定。继续推进资本市场改革开放和稳定发展。扩大企业债券发行规模。拓宽中小企业融资渠道。(7)认真落实投资体制改革决定，尽快制定和完善各项配套措施。规范企业投资项目的核准制和备案制，真正落实企业投资自主权。规范政府投资范围和行为，提高政府投资决策的科学化、民主化水平。实行政府投资项目公示制度，尽快建立政府投资责任追究制度。完善适应新形势的投资宏观调控体系，建立健全投资监管体系。及时研究解决改革过程中出现的新情况、新问题。(8)积极发展资本、土地、技术和劳动力市场。规范发展产权交易市场。大力推进社会信用体系建设。(9)继续深化价格改革，完善水、电、服务等价格形成机制。严格实施成本监审，完善政府价格决策听证制度，提高政府定价的科学性。
　　(七)努力提高对外开放的质量和水平，更好地利用两个市场、两种资源。(1)加快转变对外贸易增长方式。将外贸进出口与提升产业结构和产品结构、提高技术装备水平、培育自主研发能力结合起来。完善出口退税机制。促进加工贸易转型升级。实施名牌战略，支持和扩大具有自主知识产权、自主品牌的商品和服务出口，提高传统出口商品的质量、档次，规范外贸经营秩序。组织好国内急需和短缺的能源、原材料、关键技术和重大设备的进口。(2)着力提高利用外资质量。鼓励外商投资高新技术产业、先进制造业、现代服务业、现代农业和环保产业，参与国有企业改组改造。鼓励和吸引跨国公司来华设立研发中心、采购中心、地区总部和先进生产制造基地。稳步推进银行、保险、证券、商业、电信、旅游等服务领域的对外开放。用好国际金融组织和外国政府贷款，加强外债总量调控，优化外债结构，防范外债风险。(3)积极实施“走出去”战略。加强境外投资的规划和产业政策指导，完善财税、金融、保险、外汇等支持政策，鼓励有条件的企业到境外投资。巩固和发展多边、双边经贸关系，积极参与区域经济合作。
　　(八)认真做好就业和社会保障工作，不断提高人民生活水平和质量。(1)继续实施积极的就业政策，坚持在发展中解决就业问题，大力发展就业容量大的第三产业、中小企业和劳动密集型产业，增加就业岗位。加强政府对就业的指导、培训和服务，建立劳动就业监测预警体系。统筹做好城镇下岗失业人员新增劳动力、高校毕业生、复员转业军人的就业和农村富余劳动力的转移工作。把支持就业再就业的各项政策措施落到实处。增加各级财政支持再就业的资金投入，加快建立和完善公共就业服务体系，加大对困难地区、困难行业和困难群体再就业的支持力度。规范企业裁员行为。(2)加快建立与经济发展水平相适应的社会保障体系。继续巩固“两个确保”，力争按期完成国有企业下岗职工基本生活保障制度向失业保险制度并轨。健全社会统筹与个人账户相结合的城镇企业职工基本养老保险制度，逐步做实个人账户。提高非公有制企业和灵活就业人员的社会保险参保率。抓紧研究机关事业单位养老保险制度改革方案。有条件的地区要积极探索建立农村社会保障体系。健全城乡社会救助体系，完善和规范城市“低保”制度。完善农村“五保户”供养制度。继续抓好开发式扶贫工作，增加并切实管好、用好各项扶贫资金。(3)努力增加城乡居民尤其是中低收入群体收入。完善并严格执行城镇职工最低工资制度，督促企业按时足额支付职工工资。合理调整收入分配结构。规范分配秩序，进一步完善个人所得税制度，加强对垄断行业收入分配的调控和监管。进一步改革机关事业单位工资制度。(4)增强消费对经济增长的持久拉动作用。积极推动消费结构升级，扩大住房、汽车、文化、旅游、健身等方面的消费。实行有利于扩大消费的财税、金融和产业政策。加快建立电信普遍服务基金，推动农村通信发展。继续改善消费环境，重点整顿和规范教育、医疗服务等领域收费和价格秩序，严厉打击假冒伪劣等各种违法犯罪行为，实施好食品、药品放心工程，提倡绿色消费，加强对消费者权益的保护。大力发展现代流通方式，加快社会信用体系建设，规范发展消费信贷。
　　(九)全面促进科技教育和各项社会事业发展，努力构建社会主义和谐社会。(1)大力发展科技第一生产力。抓紧制定并实施《国家中长期科学和技术发展规划纲要》。加强基础研究、战略高技术和社会公益性技术研究，启动散裂中子源、强磁场等9项国家重大科技基础设施建设，支持原创性基础研究。再改造一批国家重点实验室。加大产业发展的共性、关键技术的攻关力度和对引进先进技术消化吸收创新的支持力度。继续组织实施电动汽车、功能基因等重大科技专项。积极推进安全生产、环保与资源勘探等10大产业技术开发。加强国家创新体系建设。深化科技体制改革。继续推进社会公益类科研机构的分类改革。支持企业创新能力建设。进一步繁荣和发展哲学社会科学。坚持不懈地做好科普工作，推动全民科学素质提高。(2)优先发展教育事业。全面实施新一轮教育振兴行动计划，促进各级各类教育协调发展。完善农村义务教育管理体制，建立健全投入保障机制。扎实推进国家西部地区“两基”攻坚计划，继续实施农村中小学现代远程教育工程和危房改造工程，健全和完善贫困家庭学生资助体系。加强职业教育和继续教育，实施中西部职业教育发展专项建设计划，加快培养紧缺人才和技能型人才。继续推进高等教育发展和改革，提高高等教育质量。(3)加快公共卫生体系建设。全面完成疾病预防控制体系和基本完成医疗救治体系建设任务。加强重大传染病、地方病特别是艾滋病、血吸虫病和肺结核病的防治。有效整合和优化配置医疗卫生资源，加强农村医疗卫生基础设施和农村卫生队伍建设，继续稳步推进新型农村合作医疗试点和农村医疗救助工作。加快开展城市医疗服务体制改革试点。(4)大力发展文化、广播影视、新闻出版、体育等事业。积极发展公益性文化，弘扬民族优秀传统文化，抓紧保护和抢救优秀的特别是濒临失传的民族文化，促进经营性文化产业健康发展。进一步加强文化设施建设和文化、自然遗产保护工作。加快国家博物馆等重大文化工程建设。大力发展“红色旅游”。继续推进广播电视“村村通”工程。加强农村计划生育服务设施建设，改善和加强流动人口管理。做好新闻出版工作。促进体育事业健康发展，抓紧2008年奥运会各项准备工作。(5)切实维护社会稳定。高度重视并认真解决征地拆迁、企业改制、水库移民、劳资纠纷等方面存在的损害群众利益的问题。妥善解决城乡困难群体的实际困难。进一步加强安全生产工作，完善安全生产管理体制和机制，增加对安全设施的投入。积极预防和妥善处置群体性事件，完善应对突发事件的应急机制。加强社会治安防控体系建设，认真做好重大自然灾害调查、监测、预报和治理工作，确保人民群众生命财产安全。
　　今年还有一项重要任务，就是编制好《国民经济和社会发展“十一五”规划和2020年远景目标》。
　　2005年我国改革、发展、稳定的任务相当艰巨和繁重。我们要在以胡锦涛同志为总书记的党中央领导下，高举邓小平理论和“三个代表”重要思想伟大旗帜，全面落实科学发展观，齐心协力，扎实工作，为实现2005年国民经济和社会发展预期目标和各项任务而努力奋斗！
　　----------------------
　　附：名词解释
　　国民经济和社会发展预期目标。是政府对年度经济和社会发展主要方面期望达到的目标。预期目标本质上是导向性的，在反映经济社会发展基本趋势的同时，主要是向社会传递宏观调控的意图，以引导市场主体行为，不等同于预测值。预期目标的确定要综合考虑国内外各种影响因素，兼顾需要和可能，适当留有余地，并可以随着经济运行环境和发展趋势的变化进行调整。在社会主义市场经济条件下，政府主要是通过运用经济的、法律的手段来促进国民经济和社会发展预期目标的实现。
　　城镇登记失业率。是城镇登记失业人员与城镇单位就业人员、城镇单位中的不在岗职工、城镇私营业主和个体户主、城镇私营企业和个体就业人员、城镇登记失业人员之和的比值。在城镇单位就业人员中，不包括使用的农村劳动力、聘用的离退休人员、港澳台及外方人员。城镇登记失业人员是指有非农业户口，在一定的劳动年龄内(16岁以上及男50岁以下、女45岁以下)，有劳动能力，无业而要求就业，并在当地就业服务机构进行求职登记的人员。
　　价格滞后影响。也称翘尾影响。是指上年商品(服务)价格上涨对下一年价格指数的影响。例如，假设食盐上年前6个月价格均为每公斤1元，而7月份价格上涨至每公斤1.2元，并一直保持到12月份。这样，即使下一年食盐价格未作调整，仍为每公斤1.2元，但下一年前6个月与上年同期相比，食盐价格将表现为上涨20%。这就是上年7月份食盐价格上涨对下一年价格指数的滞后影响。
　　货币供应量。是指某个时点上全社会承担流通和支付手段的货币存量。现阶段，我国将货币供应量划分为三个层次：一是流通中现金M0，即在银行体系外流通的现金；二是狭义货币供应量M1，即M0加上企事业单位活期存款；三是广义货币供应量M2，即M1加上企事业单位定期存款、居民储蓄存款和其他存款。
　　窗口指导。指中央银行通过劝告和建议来影响商业银行信贷行为，属于温和的、非强制性的货币政策工具。针对2004年上半年信贷投放明显偏快的势头，人民银行、银监会多次对商业银行进行窗口指导，对控制贷款总量过快增长、优化信贷结构起到了积极作用。
　　内地与香港、澳门建立更紧密经贸关系安排。为进一步加强内地与香港、澳门两个特别行政区的经贸合作关系，2003年内地与香港、澳门分别签署了关于建立更紧密经贸关系安排的协议，并于2004年1月1日起开始实施。总体目标是：逐步减少或取消双方之间所有货物贸易的关税和非关税壁垒；逐步实现服务贸易的自由化，减少或取消双方之间存在的所有歧视性措施；促进贸易投资便利化。
　　农村“六小工程”建设。包括节水灌溉、人畜饮水、乡村道路、农村沼气、农村水电、草场围栏等在内的农村中小型基础设施建设。
　　电力需求侧管理。指通过综合运用经济、技术等多种手段，鼓励和引导用户节约用电，转变用电方式，在高峰时段少用电、在低谷时段多用电，提高终端用电效率，优化资源配置，改善和保护环境，实现最小成本电力服务。同时，在供电紧张时组织供电企业有序供电，确保居民生活、农业生产及重点行业用电需求。
　　煤电价格联动机制。是为了缓解当前煤电价格矛盾而拟采取的一项措施。国务院批准的《关于建立煤电价格联动机制的意见》规定，以不少于6个月为一个周期，当一个周期内电煤价格变动幅度超过上一周期的5%时，在电力企业消化煤价变化因素30%的基础上，相应调整上网电价，并按电网经营企业输配电价保持相对稳定的原则，调整销售电价。其中，居民生活用电、农业用电、中小化肥企业用电价格一年只能调整一次。
　　差别电价。为抑制高耗能行业盲目发展，促进技术进步和产业结构升级，根据国家产业政策，按照能耗、物耗、环保、技术装备水平等，将电解铝、铁合金、电石、烧碱、水泥、钢铁等6个高耗能行业的工艺装备、技术和产品划分为允许和鼓励类、限制类、淘汰类三类。对限制类和淘汰类的用电执行相对较高的销售电价。
　　现代物流。是融合运输业、仓储业、信息业、批发零售业、对外贸易业等形成的新兴复合型服务产业。其核心是按照系统整合的理念，运用信息技术对分散的运输、装卸、包装、信息、流通加工、储存等环节的资源进行整合，实行一体化运作，以降低成本、提高效率、优化服务。
　　循环经济。指以资源的高效利用和循环利用为核心，以低消耗、低排放、高效率为基本特征的一种经济增长模式。循环经济的基本原则是“减量化、再利用、资源化”。所谓“减量化”，是指在生产和服务过程中，尽可能地减少资源消耗和废弃物的产生，核心是提高资源利用效率。所谓“再利用”，是指产品多次使用或修复、翻新或再制造后继续使用，尽可能地延长产品的使用周期，防止产品过早地成为垃圾。所谓“资源化”是指废弃物最大限度地转化为资源，变废为宝、化害为利，既可以减少自然资源的消耗，又可以减少污染物的排放。
　　节约型社会。是指在社会再生产的生产、流通、消费诸环节中，通过健全机制、调整结构、技术进步、加强管理、宣传教育等手段，动员和激励全社会节约和高效利用各种资源，以尽可能少的资源消耗，满足人们不断增长的物质文化需求。它是以较低资源消耗支撑全社会较高福利水平的可持续的社会发展模式。
　　“三河三湖”等重点区域水污染防治工程。“三河三湖”是指辽河、海河、淮河和太湖、巢湖、滇池。“九五”期间，国家陆续批准了“三河三湖”等水污染严重地区的流域污染防治规划，提出了阶段性工作目标，并安排了国债专项资金。目前，重点区域水污染防治工程范围除“三河三湖”外，还包括渤海、三峡库区、南水北调东线等，下一步拟扩展到松花江、黄河、长江等重点流域。
　　资源开发补偿机制。指为了实现资源型城市可持续发展，在资源开采的不同阶段(依资源开发程度可分为成长期、鼎盛期和衰退期)，通过采取建立资源开发补偿资金等一整套补偿措施和扶持办法，支持矿产资源勘探与合理开发、保护和恢复被破坏的地质环境和生态环境以及经济转型。
　　衰退产业援助机制。指通过财税扶持、资金和项目支持、减轻企业负担等方式，对伴随着资源的减少而走向衰退的产业进行直接援助，或通过政策引导和支持发展接续产业进行间接援助，促进资源型城市经济转型。
　　战略高技术。主要是指具有前沿性的，一旦在技术上取得突破将对整个技术进步和高技术产业发展产生巨大影响和带动作用，事关国家全局和长远利益，并对国家经济、社会、安全和综合国力具有战略影响的高技术或高技术群。
　　“两基”人口覆盖率。指一个地区按照国家规定的有关验收标准，已实现“基本普及九年义务教育、基本扫除青壮年文盲”的县(包括县级行政单位)的人口数之和占本地区总人口数的比重。2002年、2003年全国“两基”人口覆盖率分别为91%和91.8%。2004年国务院批准实施《国家西部地区“两基”攻坚计划》，目标是到2007年底在西部地区实现基本普及九年义务教育，基本扫除青壮年文盲。
　　新一轮教育振兴行动计划。即2004年国务院批转的《2003－2007年教育振兴行动计划》。主要包括两大战略重点和六项重大工程。两大战略重点是，继续把农村教育放在“重中之重”的地位；努力建设若干所世界一流大学、一批国际知名的高水平研究型大学以及一批重点学科。六项重大工程分别是：新世纪素质教育工程、职业教育与培训创新工程、高校教学质量与教学改革工程、促进毕业生就业工程、教育信息化建设工程、高素质教师和管理队伍建设工程。
　　农村中小学现代远程教育工程。2003年国务院决定实施农村中小学现代远程教育工程。主要内容是，用5年左右时间，安排中央专项资金50亿元，加上地方配套资金，在农村小学教学点基本配备光盘播放系统，在农村小学基本配备卫星教学收视点，在农村初中基本配备计算机教室。目的是通过现代信息技术手段，输送优质教育资源，提高农村中小学师资水平和教育质量。
　　公共卫生体系。公共卫生是政府主导，社会共同参与，通过改善环境卫生条件，提供基本医疗卫生服务，倡导良好卫生习惯和文明生活方式等措施，预防和控制传染病及其他疾病流行，促进和保障人群健康的社会公益事业。我国的公共卫生体系包括疾病预防控制体系、突发公共卫生事件医疗救治体系、农村卫生体系、城市基本医疗服务体系、环境卫生体系、卫生执法监督体系等。
　　广播电视“村村通”工程。指从1998年开始国家组织实施的扩大农村地区广播电视覆盖面的工程。截至到2002年，基本实现了已通电行政村通广播电视目标，解决了7000多万农民群众收听收看广播电视问题。目前，正在开展中部地区国家扶贫工作重点县和西部地区规模较大的已通电自然村的“村村通”工程建设，共涉及8万多个自然村。
　　投资项目核准制。核准制是指对企业投资建设不使用政府性资金的重大项目和限制类项目不再由政府进行审批。政府只是从维护经济安全、合理开发利用资源、保护生态环境、优化重大布局、保障公共利益、防止出现垄断等方面进行核准。项目的市场前景、经济效益、资金来源和产品技术方案等均由企业自主决策、自担风险，但要依法办理环境保护、土地使用、资源利用、安全生产、城市规划等许可手续。实行核准制的范围和权限，由《政府核准的投资项目目录》作出规定。
　　投资项目备案制。备案制是指企业投资建设不使用政府性资金的非重大项目和非限制类项目，由企业按照属地原则向地方政府投资主管部门备案后，依法办理环境保护、土地使用、资金利用、安全生产、城市规划等许可手续。其后，企业即可自行组织建设。企业报送备案的项目，除不符合法律法规有关规定、产业政策禁止发展、需报政府核准或审批的之外，地方政府投资主管部门应当予以备案。

© 北大法宝：（[www.pkulaw.com](https://www.pkulaw.com/)) 专业提供法律信息、法学知识和法律软件领域各类解决方案。北大法宝为您提供丰富的参考资料，正式引用法规条文时请与标准文本核对。
欢迎查看所有[产品和服务](https://www.pkulaw.com/helps/2.html)。法宝快讯：[如何快速找到您需要的检索结果？](https://www.pkulaw.com/helps/70.html)[法宝](https://www.pkulaw.com/helps/69.html)V6有何新特色？

扫描二维码阅读原文

**原文链接：**<https://www.pkulaw.com/chl/88dbb277e6113858bdfb.html>
